# Children of Nature: Thoughts on Targeted and Untargeted Analytical Approaches to Decipher Polyphenol Reactivity in Food Processing and Metabolism

**DOI:** 10.1021/acs.jafc.3c09211

**Published:** 2024-08-05

**Authors:** Nikolai Kuhnert

**Affiliations:** School of Science, Constructor University, Campusring 8, 28759 Bremen, Germany

**Keywords:** polyphenols, mass spectrometry, untargeted metabolomics, coffee, tea

## Abstract

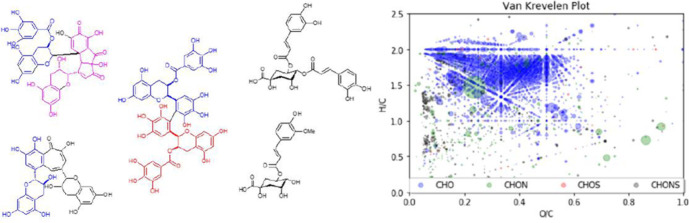

Following 25 years of polyphenol research in our laboratory, the astonishing chemical and metabolic reactivity of polyphenols resulting in considerable chemical diversity has emerged as the most remarkable attribute of this class of natural products. To illustrate this concept, we will present selected data from black tea and coffee chemistry. In black tea chemistry, enzymatic fermentation converts six catechin derivatives into an estimated 30 000 different polyphenolic compounds via a process we have termed the oxidative cascade process. In coffee roasting, around 45 chlorogenic acids are converted into an estimated 250 novel derivatives following a series of diverse chemical transformations. Following ingestion by humans, these dietary polyphenols, whether genuine secondary metabolites or food processing products, encounter the microorganisms of the gut microbiota, converting them into a myriad of novel structures. In the case of coffee, only two out of 250 chlorogenic acids are absorbed intact, with most others being subject to gut microbial metabolism. Modern mass spectrometry (MS) has been key in unravelling the true complexity of polyphenols subjected to food processing and metabolism. We will accompany this assay with a short overview on analytical strategies developed, including ultrahigh-resolution MS, tandem MS, multivariate statistics, and molecular networking that allow an insight into the fascinating chemical processes surrounding dietary polyphenols. Finally, experimental results studying biological activity of polyphenols will be presented and discussed, highlighting a general promiscuity of this class of compounds associated with nonselective protein binding leading to loss of enzymatic function, another noteworthy general property of many dietary polyphenols frequently overlooked.

## Introduction

Following my first academic appointment at the University of Surrey 25 years ago, I met Mike Clifford, who introduced me to the world of polyphenol chemistry, therefore newly defining my research interests for the last two decades. At this point in time, roasted coffee and black tea chemistry was considered a “terra incognita”, with a plethora of polyphenolic compounds formed during food processing by thermal treatment or fermentation and a complete lack of understanding of structures formed or reaction mechanisms defining this type of chemistry. I was trained as a synthetic organic and natural product chemist and initially less productive approaches focused on synthesis. In 2003, we purchased our first ESI ion trap mass spectrometer (MS), which allowed us immediate insight into coffee chemistry.^1^ With first ultrahigh-resolution MS measurements in Bremen in 2008, the world of black tea chemistry^2^ opened up followed by insight into the composition of many more processed food materials.

Consequently, my research group studied the chemistry of dietary polyphenols in food processing and recently moving as well to human metabolism. This essay will focus on two rather neglected crucial side-aspects of polyphenol chemistry, constantly emerging whatever the processed food analyzed and discuss them with examples from coffee and black tea chemistry. These are

- Polyphenols are highly reactive in chemical and enzymatic transformations (both in metabolism and food processing).
- Polyphenols are predisposed to furnish chemical diversity.
- Polyphenols show biological promiscuity mostly by nonselective protein binding.
- Nevertheless, polyphenols from the diet or medicinal plants are biologically active and beneficial to human health, but we do not fully understand how and why!

Plant polyphenols are secondary plant metabolites produced by all terrestrial plants. Second to carbohydrates, they are the most abundant organic molecules on our planet.^3^ If glycosylated phenolics are considered as phenolics and not as carbohydrates, they are even the most abundant class of molecules on our planet. Plants produce small molecule polyphenols of several subclasses (classified according to chemical structure or properties tannins, hydroxycinnamates, flavonoids, etc.), which are further transformed into oligomeric structures or incorporated into polymeric structure, mainly lignins.^4^ Furthermore, senescing plant tissue upon degradation produces humic acid, an important further class of phenolic material, considerably adding to the total mass of polyphenolics on our planet.^5^ Natural product chemists have been interested in polyphenolics for decades, focusing on their structure, chemical properties, and biological activities. The real surge of interest in polyphenol chemistry arose with the realization of their importance for the human diet.^6,7^ Humans consume large amounts of polyphenols with their daily diet. The exact quantity of polyphenol intake is hard to estimate because all figures depend on the definition of polyphenols, availability of quantitation methods, and the accuracy of the available data.^8,9^ A beneficial effect for human health arising through high polyphenol consumption must be accepted beyond all doubt due to countless epidemiological and clinical intervention studies and must be considered as scientific consensus. It is worth noting that most pharmacopoeias contain a selection of polyphenol rich herbal drugs obtained from medicinal plants grouped in flavonoid drugs such as *Crategi folium*, *Ginkgo biloba*, *Betulae folium*, *Viola tricoloris herba*, or *Sambucci flos*, along with polyphenol rich tannin drugs such as *Quercuscortex*, *Myrtilli fructus*, or *Juglandis folium* used to treat a variety of diseases. Due to the complexity of polyphenol chemistry, the question of which compound or group of compounds is responsible for a beneficial health effect remains mostly open. Similarly, the mechanism of action, following consensual criticism of the antioxidant hypothesis,^10,11^ and biological target of polyphenols, remain largely elusive, requiring a joint multidisciplinary effort to provide a better understanding leading ultimately to sound dietary advice to the consumer as a long-term goal.

**Figure 1:**
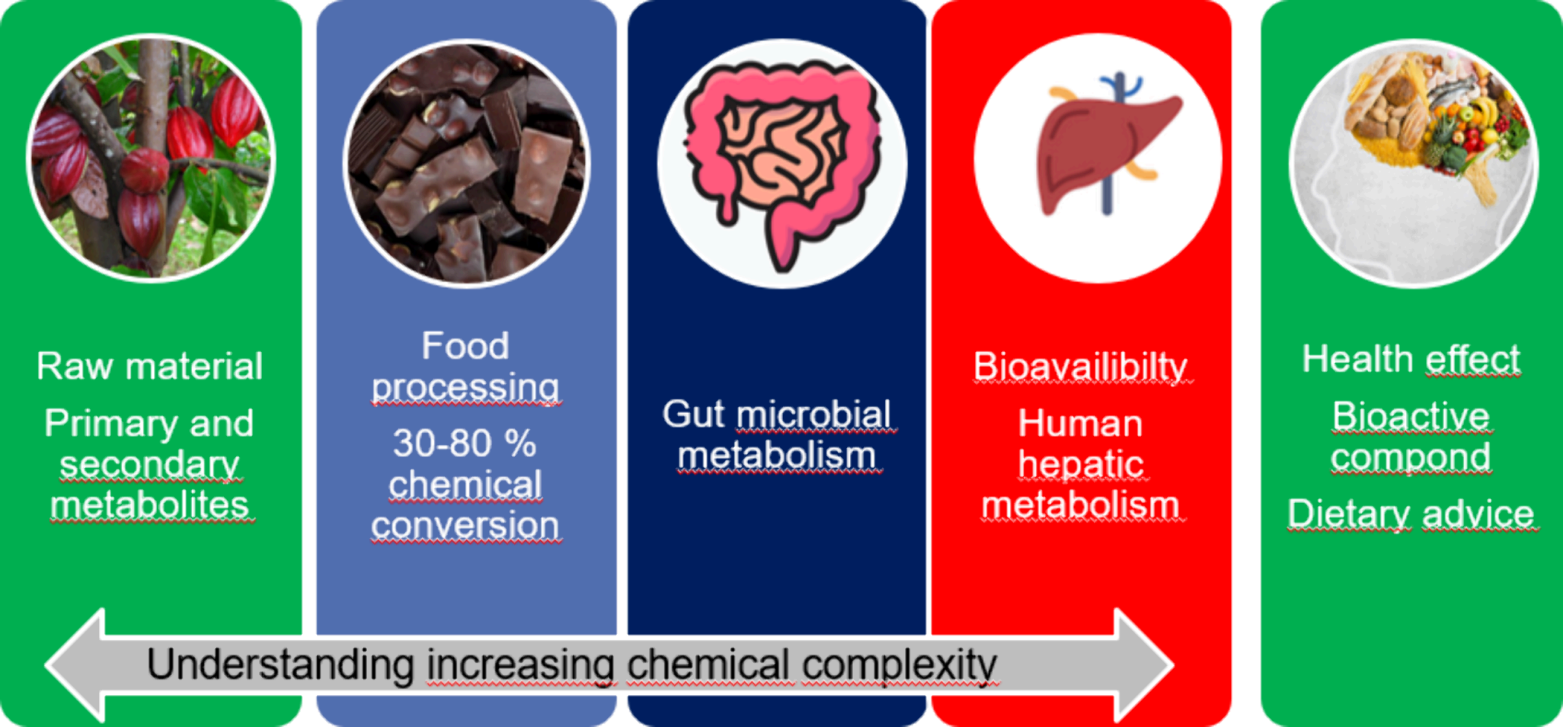
Schematic figure showing origin of biologically active compounds in the diet from secondary plant metabolites to food processing compounds, gut microbial metabolites, and human hepatic metabolites.

For identification of the biologically active compound, the full chain of events from the product to human metabolism must be considered. Biologically active polyphenols could be genuine secondary plant metabolites, could arise from food processing, could be derived from gut microbial metabolism of secondary metabolites of food processing products, or finally could arise from human liver metabolism (see Figure 1). Edwin Haslam has coined the term “Children of Nature”^12^ initially for plant secondary metabolites and in later publications, including as well compounds formed by processing or formed due to their inherent reactivity.^13^ Hence, I propose to refer to all polyphenol derivatives formed by processing and metabolism as such Children of Nature. In each step, polyphenolics undergo intense chemical transformations due to their inherent reactivity, and the complexity of the polyphenol chemical community is increasing with each step both in the numbers of compounds formed and in the chemical complexity of structures obtained. Understanding aspects of this chemistry has been a true analytical challenge and the focus of our research endeavors, and we will briefly summarize and discuss some facets of this work.

## Targeted and Untargeted Analysis Based on Mass Spectrometry

In the contemporary world of analytical chemistry and the age of Omics, mass spectrometry based analytical strategies are often divided into targeted and untargeted approaches. In my use of the terms, targeted analysis refers to analysis where authentic reference standards are available and the analytic workflow specifically searches for these available reference molecules. Such authentic reference standards can be obtained by organic synthesis or following preparative isolation from natural samples. In food processing and human metabolism, a structural hypothesis of the target molecule is required prior to its synthesis. As a consequence, structural assignment is carried out beyond doubt and absolute reliable quantitative data are obtained. As a practical alternative to synthesis, expanding the scope of targeted analysis, I would like to advocate as well the use of surrogate standards, whereby a natural source containing previously well characterized compounds can serve as a starting point for targeted analysis.^14^

In untargeted analysis, all detectable species are recorded and an attempt to annotate chemical structures follows. The most common procedure involves comparison to MS libraries for dereplication. If this approach is unsuccessful due to a lack of database entries, manual data interpretation follows for which several “inspirational” and facilitating tools and approaches have been developed by us and others.

**Figure 2:**
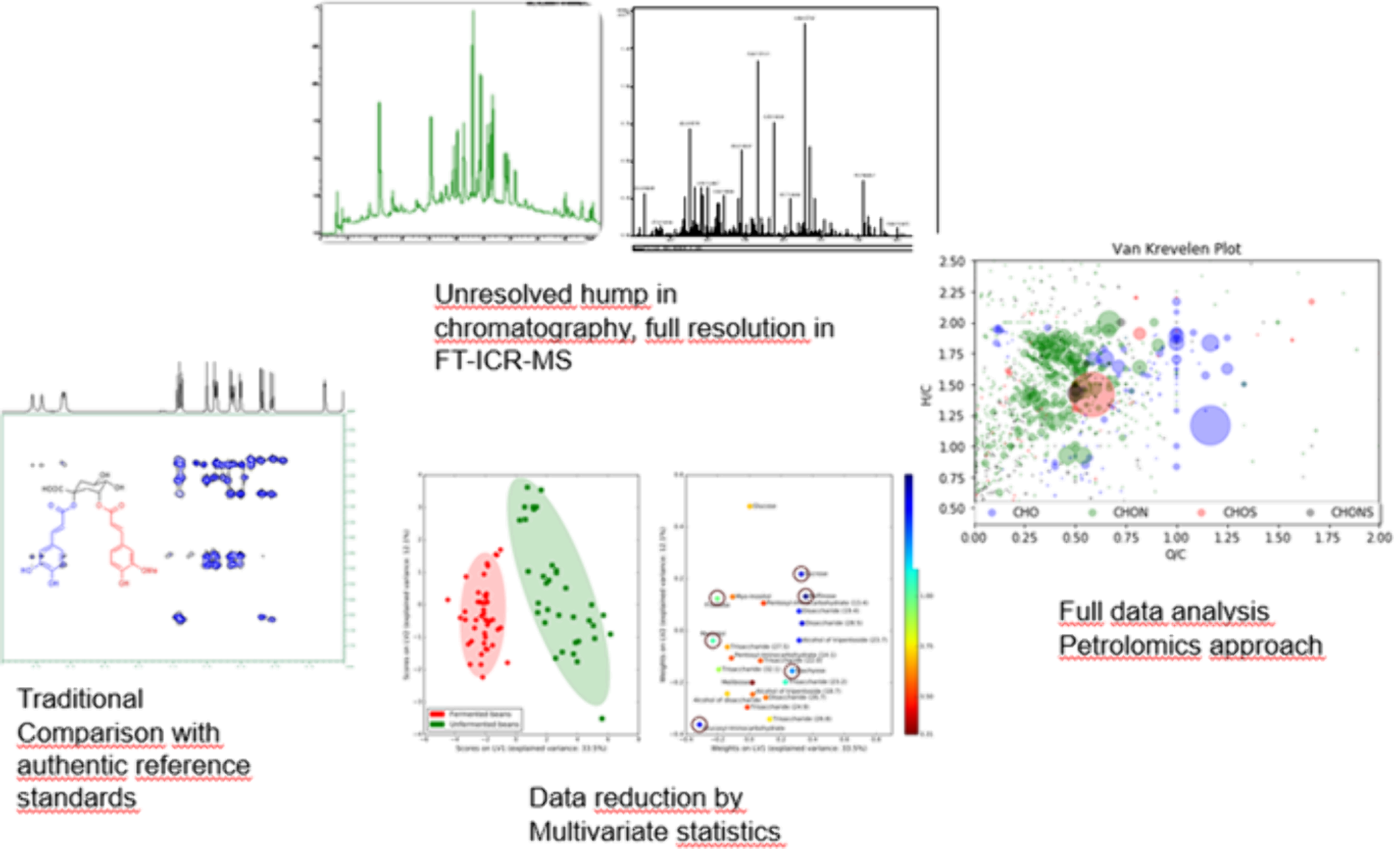
Schematic representations of analytical strategies addressing unresolved complex mixtures from food processing or human body fluids showing targeted analysis with authentic standards, data reduction, multivariate statistics, and untargeted “petrolomic-style” ultrahigh-resolution mass spectrometry.

In the field of food processing and human metabolism, in most samples thousands or even tens of thousands of compounds can be readily detected by mass spectrometry. In a chromatographic analysis, typically, a hump is apparent due to the many compounds that cannot be chromatographically resolved. This phenomenon has been termed an unresolved complex mixture (UCM). In general, three approaches are available to address such analytical challenges, schematically shown in Figure 2: (1) targeted analysis using authentic standards, (2) data reduction using multivariate statistics, or (3) untargeted analysis using high resolution and tandem MS. For a minute fraction of these authentic standards, are available, and for a slightly higher number of compounds MS library entries are available. Realistically, however, these cover at best 10–20% of the number of compounds encountered in a typical sample of let us say black tea, cocoa powder, or red wine or even human body fluids. The number of compounds here refers to the number of good quality MS/MS spectra obtained from a given LC-MS chromatographic analysis. Concomitantly, structures formed are chemically complex, with multiple stereogenic centers combined with complex arrays of functional groups posing a true challenge for organic synthesis. Thus, it is inconceivable that organic synthesis will solve this challenge due to a lack of funding and resources. If we take the estimated 30 000 compounds contained in black tea, thousands of skilled synthetic chemists would need to spend their working life achieving the synthesis of all authentic standards required. Untargeted approaches with only tentative structures as a consequence are the only realistic alternative. In a recent essay, we have discussed this dilemma and compared it to Ulysses’s choice between Scylla and Charybdis.^15^ If we rely on authentic standards only, progress and increase of scientific knowledge and insight will be minimal; scientific progress will be almost frozen in time. If we embrace the untargeted world of tentative structures, false positive annotations from MS libraries or incorrect tentative structure assignments will be commonplace, however, will result in an increase of our knowledge and scientific progress. An important adjustment to common practice on this route would be a publication system, similar to Wikipedia, that allows rectification of erroneous structure assignments.

## Untargeted Analysis by Ultrahigh-Resolution MS

For understanding black tea chemistry, we had a real scientific breakthrough when first employing ESI-FT-ICR-MS methods. In typical black tea samples, an excess of 10 000 peaks could be resolved in a single experiment.^16^ Hence, this method gives a direct count of all species detectable under given ionization conditions. The number must be multiplied by the average number of isomers present because the method is isomer-blind. How many compounds escape detection remains uncertain. Numbers for other processed foods are summarized in a review.^17^ These 10 000 MS signals can be directly converted into a list of thousands of molecular formulas. From these formulas, elemental ratios such as H/C, O/C, or N/C can be calculated and displayed on a two-dimensional plot termed the van Krevelen plot.^18^ The beauty of this plot lies in the fact that most classes of natural products and their derivatives have clearly defined boundaries for elemental ratios, allowing a rough classification of classes of compounds present. For example in a black tea sample, 90% of observed peaks could correspond to polyphenols,^19^ whereas in cocoa, 80% of signals could correspond to small peptides and their derivatives.^20^

Second, the Kendrick formalism, introduced by Marshall,^21^ allows identification of reactivity patters within the compounds formed in a sample. Using the mass defect formalism, homologous series of compounds can be identified in a sample whereby the term homologous series refers to a group of compounds, in which starting with a parent structure multiple identical moieties are added. As an unpublished example, Figure 6 contains such a plot showing a homologous series of thea naphthoquinones with successive addition of oxygen atoms.

## Untargeted Analysis by Tandem Mass Spectrometry

Tandem mass spectrometry is an incredibly powerful tool, allowing at times full structure elucidation akin to NMR spectroscopy. Most of the time, it allows the formulation of a structure hypothesis with only a few structural alternatives possible. In chlorogenic acid chemistry, we could show that regio-isomeric derivatives show unique fragment spectra that allow for an unambiguous assignment of structures. In follow-up work, we extended this approach to caffeoyl glucoses,^22^ shikimates,^23^ diastereoisomers of quinic acid,^24^ and others.

When interpreting a tandem MS data set, several useful approaches deserve to be mentioned here. Parent structures can be readily searched by creating extracted ion chromatogarms in MS^2^. For example, all caffeoyl quinic acids (CQAs) show a fragment ion at *m*/*z* 353 or all feruloyl quinic acids at *m*/*z* 367.^25^ Similarly a neutral loss search allows for identification of all structures with a common moiety lost in fragmentation. Common examples include loss of 162 Da (hexoside), 132 Da (pentoside),^26^ 43 Da (acetate), and so on. Such searches reveal partial structures of molecules present. Further, it should be kept in mind that any MS^3^ spectrum of a precursor ion in MS^2^ can be assigned by mapping the MS^3^ fragment spectrum to a library entry. For example, the fragment ion at *m*/*z* 353 of 4,5-dicaffeoyl quinic acid will fragment to yield in MS^3^ a spectrum identical to 4-caffeoyl quinic acid.^27^

A incredibly powerful new method termed the tandem MS molecular networking has added immense capabilities to the annotation of tandem MS data sets.^28^ Here, fragment spectra are grouped in clusters according to their similarity. As a basic assumption, compounds with similar fragment spectra are similar in structure. If it is possible to assign with certainty one structure within a given cluster, inspiration for all other members of the cluster follows automatically. As a hint, we found it incredibly useful to add data of surrogate standards to such data sets to facilitate annotation.^29^ For example, addition of a green coffee LC-tandem MS data set to any other data set automatically identifies all chlorogenic acid clusters in a given sample under investigation.

## Untargeted Analysis by Data Reduction and Multivariate Statistics

Processed food and human body fluid samples are highly complex, containing thousands or tens of thousands of analytes. Ultrahigh resolution MS gives a comprehensive and complete overview of all species detectable, however, resulting in excessive redundant information. In most cases, the scientific question at hand clearly defines that only a few compound in the unresolved complex mixtures are relevant. Data reduction by multivariate statistics allows identification of candidate compounds that are relevant for a given scientific question such as, What are the human metabolites after coffee consumption? Which compounds define bitterness in coffee? Which compounds contribute to cocoa color? Which compounds form during cocoa fermentation and indicate a successful fermentation? The candidate compounds can be compared to molecular networks^29^ or subjected to constraints such as *T*_max_ or dosage from human body fluid data.^30^

Over the years, we have settled for three distinct multivariate statistical tools. Principle component analysis (PCA), an untargeted technique, is giving an overview on general differences between samples. Hierarchical clustering is giving an overview on similarities of samples. If two groups are directly compared and the chemical differences between groups of samples need to be established, partial least-squares differential analysis (PLSDA) provides a most important compound list of all species distinguishing samples. Here, to obtain meaningful information, normalization and scaling routines and the amount of data fed into the algorithm constitutes the most important parameter to consider in practice.^31^ Correlation networks allow identification of constituents potentially related to a given property of the sample.^32^

## Chemical Reactivity in Food Processing

Polyphenols are highly reactive chemical entities. They can be easily oxidized to form stabilized radical intermediates that consequently yield quinone structures. While the polyphenolic compound is nucleophilic by nature due to its electron donating oxygen substituents, quinone structures are electrophilic. Cinnamoyl moieties in hydroxycinnamates additionally display electrophilic character reacting with other nucleophilic species present in the food matrix during processing. Other functional groups present additionally contribute to overall reactivity. Consequently, we consider polyphenols as chemically predisposed, due to their inherent reactivity, to react under appropriate food processing or metabolic conditions to yield different and more complex structures.

## Chlorogenic Acids and Coffee Roasting

**Figure 3:**
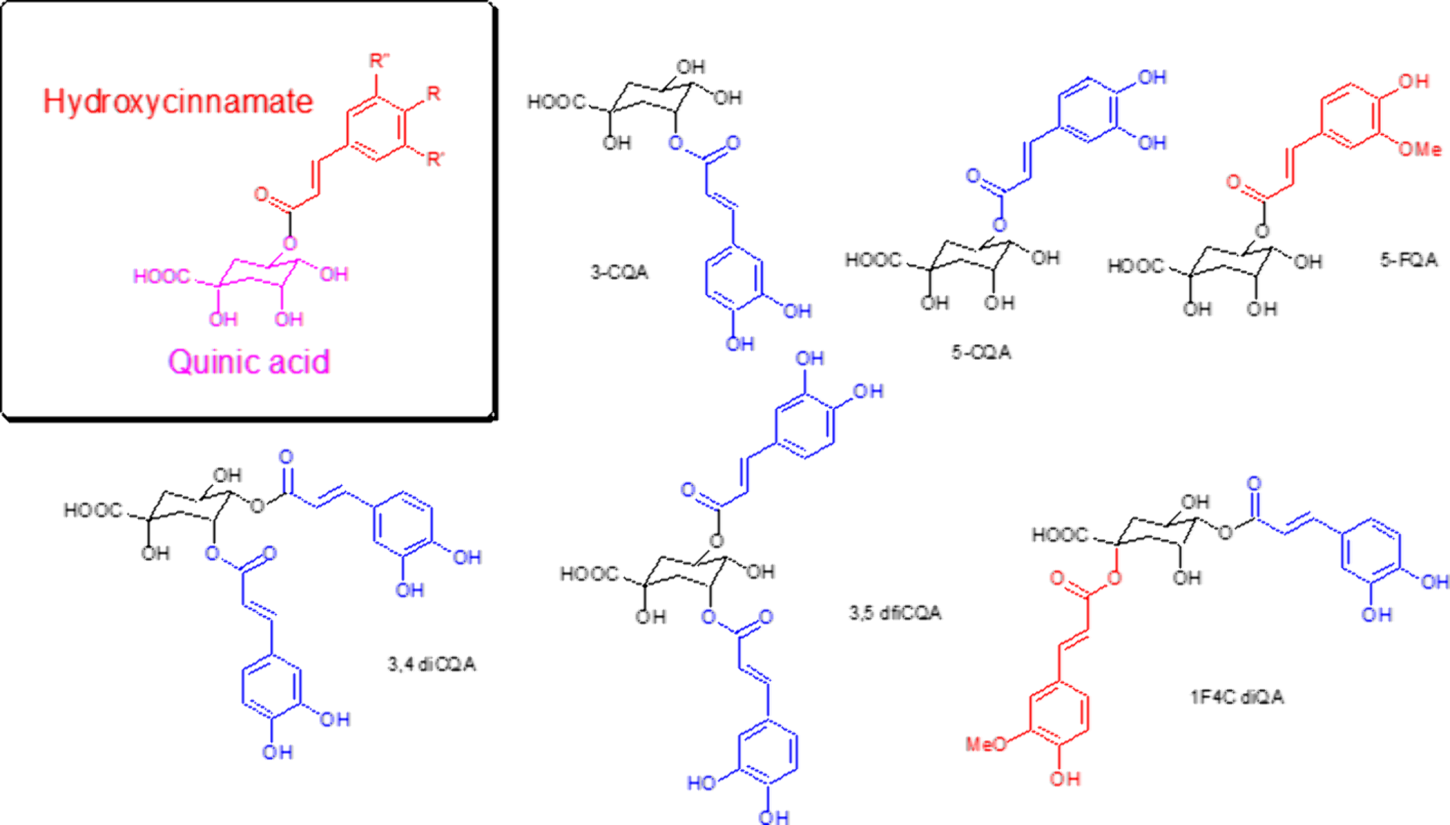
Chemical structures of selected chlorogenic acids from coffee.

Chlorogenic acids (CGAs) are by definition esters between quinic acid and hydroxycinnamic acids.^33^ Due to the nonequivalence of the four hydroxy groups in quinic acids, multiple regioisomers are possible, e.g., 4 regioisomers for monocaffeoyl quinic acid (CQA) and six for dicaffeoyl quinic acid (diCQA). Figure 3 shows some selected structures. Additionally, during food processing, *cis*-cinnamates are formed and epimers of quinic acid increased the number of isomeric structures present. CGAs must be considered as the most relevant and abundant polyphenols in our diet, with an estimated daily intake of 1–1.5 g per day per human with each cup of coffee as the major source contributing 200 mg.^34^ In Arabica green coffee beans, we observed a total of 45 different CGA isomers, whereas Robusta coffee produces around 80 compounds as sets of isomers with different cinnamate substituents.^23^ Following roasting of Arabica coffee beans, the number of CGAs present increases to around 200–250.^35^

**Figure 4:**
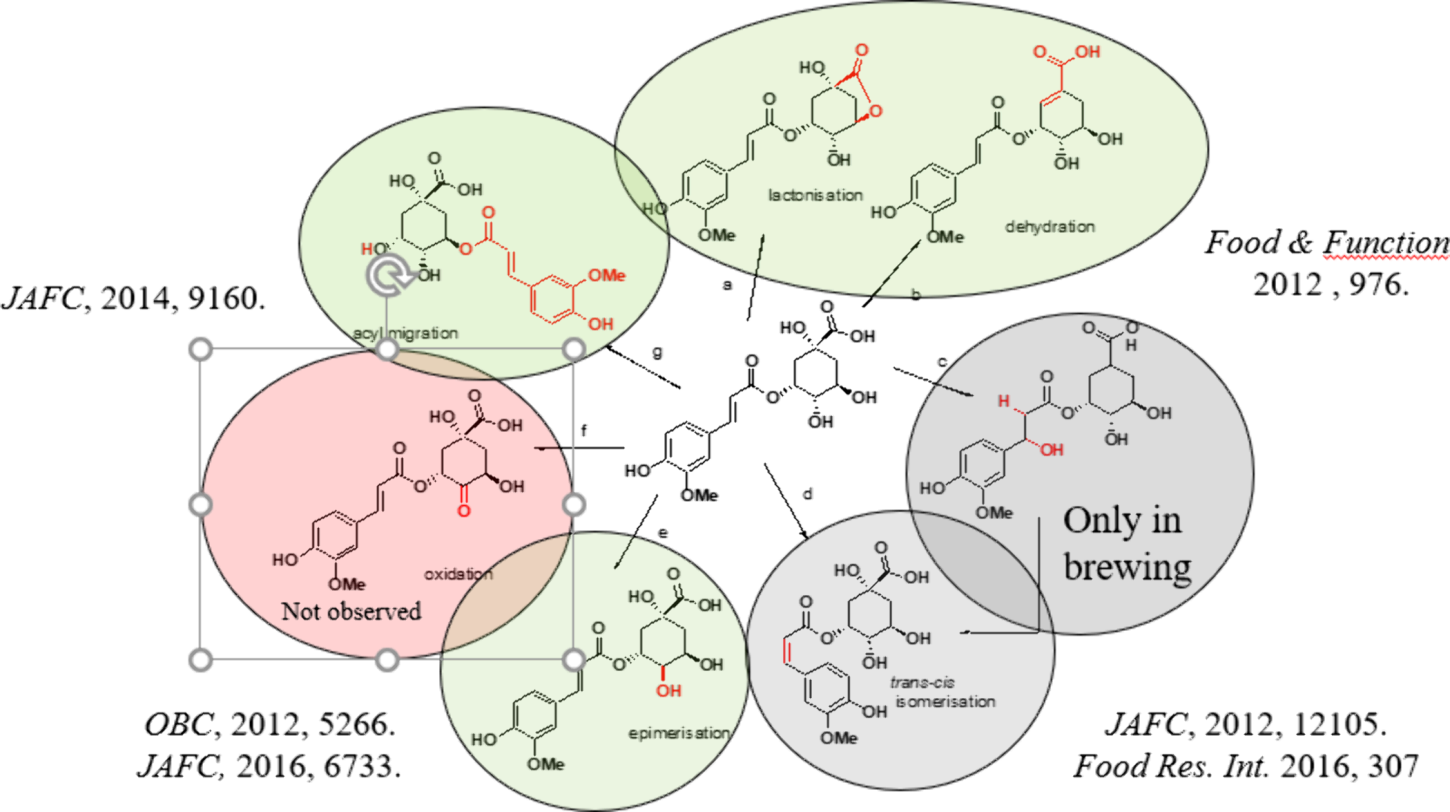
Reaction mechanisms and products of chlorogenic acids identified for coffee roasting: (a) lactonization, (b) water elimination, (c) conjugate addition of water, (d) *trans*–*cis* isomerization, (e) epimerization at quinic acid moiety, (f) oxidation at quinic acid moiety, and (g) acyl migration.

We solved the structural complexity of CGAs mainly by targeted analysis following organic synthesis of around 100 different derivatives, we assumed could form under thermal roasting conditions. Serendipitously, tandem MS data allow unambiguous identification of chlorogenic acid regioisomers^36^ and in MS^*n*^ even identification of the stereochemistry of the quinic acid base moiety.^37^ Following these investigations, we are today able to assign around 200 CGA derivatives in roasted coffee infusions. They form by a selection of five chemical reactions, transesterification leading to acyl migration,^38^ epimerization at the quinic acid moiety, dehydration to form lactones,^39^ or cyclohexene derivatives, nucleophilic addition to the cinnamoyl moiety, and subsequent β-elimination to yield *cis*-cinnamic acids.^40^ The reactions are summarized in Figure 4.

We would like to highlight the transesterification reaction, because as a reversible process it allows equilibrations of different structures. In the presence of a biological target, an isomer showing the highest binding affinity could be selected and amplified, following the Sanders concept of dynamic combinatorial chemistry.^41^ Indeed, we have experimentally observed such processes of CGAs in the presence of dairy proteins or amylases.

## Black Tea Polyphenols and Thearubigins

**Figure 5:**
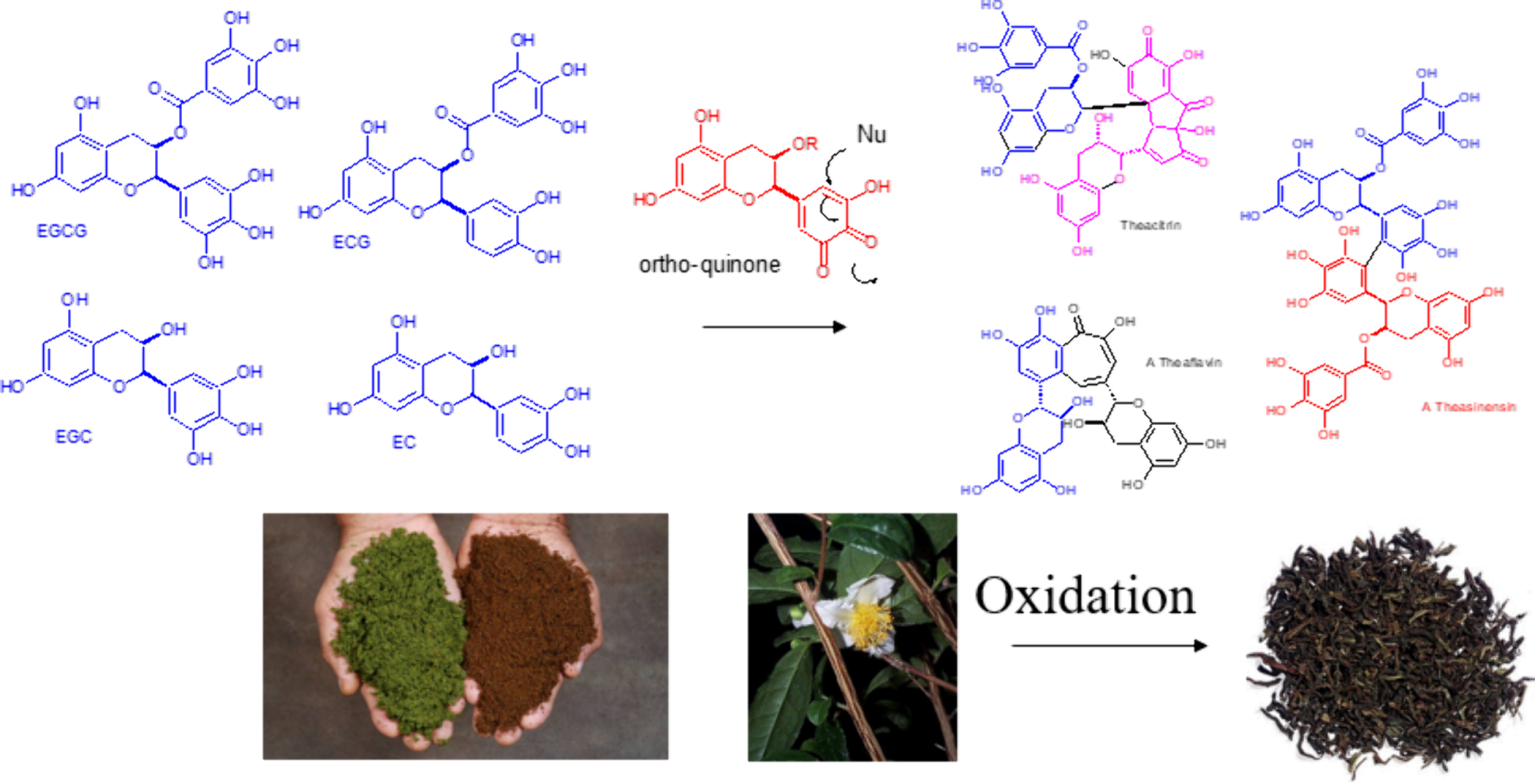
Schematic representation of tea “fermentation” leading to oxidation of green tea catechins via dimeric structures.

Green tea leaves are converted to black tea by a process termed fermentation, which in practice constitutes an enzymatic process in the absence of microorganisms. The catechins in green tea leaves are stored in the vacuoles of the cell and come into contact with a Cu-containing enzyme tea polyphenoloxidase (TPPO) following mechanical disruption of the cell membranes. Catechins serve as substrates for TPPO and are converted via quinone intermediates at the catechin B-ring to a series of dimeric structures including theaflavins, theasinensins, theacitrin, or theonaphthoquinones^42^ (see Figure 5) next to formation of a reddish material that was termed the thearubigins (TRs) by E. A. H. Roberts.^43^ The nature of this material remained elusive for decades and led to many speculations. Finally, our experiments using ESI-FT-ICR-MS revealed that the TR fraction shows 10 000 resolved signals in MS and is composed from around 5000 compounds, excluding isomers.^44^ In later work, we estimated the average isomer number as six, resulting in an estimated total of 30 000 polyphenolic compounds present in a black tea beverage.^45^ For the formation of TRs, we suggested a reaction model termed the oxidative cascade hypothesis. Following formation of dimeric catechins, further oxidation can yield oligomers with up to six catechin building blocks and connectivities of the theaflavins, theasinensins, theacitrin, or theonaphthoquinones type. In a second step, again, orthoquinones are formed which react with most abundant nucleophile in the green tea leaf and water. As a consequence, formally an aromatic CH bond is replaced by a phenolic OH moiety, hence oxygen is formally inserted into a CH bond. Multiple insertion can take place. With each additional OH the aromatic ring turns more electron rich and is easier to oxidize to produce quinones, hence the term cascade.^45^ Once the process starts with an initial enzymatic oxidation, at a threshold point, oxygen takes over as oxidizing agent and drives the process. Polyhydroxy compounds are finally in equilibrium with their quinone counter parts. We developed this scheme by initially using ultrahigh- resolution MS data with van Krevelen and Kendrick formalism and at a second stage demonstrated its validity by tandem MS experiments searching neutral losses and fragment ions expected for the hypothetic structures.

**Figure 6:**
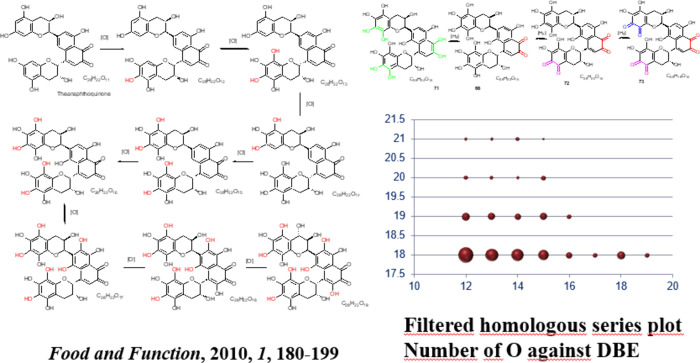
Example for oxidative cascade process in black tea fermentation for theonaphthoquinones. Kendrick plot shows homologous series of compound family with successive addition of oxygen atoms. Bubble size corresponds to chromatographic area under the peak.

Figure 6 illustrates the style showing the stepwise oxidation of a theonaphthoquinone.

## Chemical Reactivity in Human Metabolism

Following ingestions of coffee, black tea, or cocoa, the majority of polyphenols show negligent bioavailability and pass through the small intestine into the colon. Here they encounter the gut microbiota, around 1000 different bacterial species, able to take up polyphenols and metabolize them^46^ to derivatives with an increased bioavailability.^47,48^ The most commonly encountered chemical transformations of the gut microbiota include ester hydrolysis, reduction of double bonds, alkylation, and oxidative degradation of alkyl chains. Examples of highly specific reactivity patterns have also been reported with urolithin^49^ formation from ellagitannins as a prime example.^48^ Once in circulation, these derivatives are in turn subject to hepatic phase I and phase II metabolism. Again, we observe that dietary polyphenols are highly reactive as substrates to both bacterial and human metabolic enzymes, producing a myriad of metabolites. In the last three years, we have carried out three human volunteer studies, with an aim to identify polyphenol derived metabolites in human body fluids using our untargeted MS approaches. Here, we first observe that using a targeted approach, the vast majority of polyphenols cannot be detected in urine, and they are fully transformed into metabolites. For metabolites identification currently, there are four approaches possible. First, targeted analysis using authentic standards of putative metabolites as exemplified by the excellent work of the Crozier and Barron group in coffee chemistry.^50^ Second, following the pharmaceutical industry approach to metabolite hunting, based on known enzymatic reactivities, lists of theoretically possible metabolites are generated and searched.^51^ In our work, we introduced two additional approaches. We employed tandem MS molecular networking to group urinary metabolites into clusters of compounds with similar fragment spectra, following assignment of clusters to basic known dietary phenol structures, thus identifying in particular phase II derivatives of cocoa phenolics.^29^ Second, we employed multivariate statistics to identify human polyphenol metabolites from coffee^30^ and cocoa, with special constraints built into the human volunteer study design.^52^

Despite all the chemical complexity of polyphenols, I like to remind all researchers in the field to strictly follow nomenclature recommendation in order to avoid another level of confusion and complexity.^53^

## Biological Promiscuity

Many years ago, when attending my first ICPH conference, I wanted to learn as a chemist more about the biological activity and clinical applications of polyphenols. I left the conference utterly confused because I learned that common polyphenols such as quercetin, epicatechin, EGCG, resveratrol, and the like act on multiple biological targets and have multiple health benefits, in strict contrast to what I teach in my medicinal chemistry classes with biological selectivity as a unsurmountable prerequisite for any drug-like molecule. When delving into the literature, my first impression was exacerbated. Most introduction sections in polyphenol science start with long lists of possible beneficial health effects (antiobesity, antidiabetes, antioxidant, anti-inflammatory, anticancer, etc.) and at times long lists of potential biological targets. A look into search engines further reveals that compounds such as EGCG or chlorogenic acids bind to dozens and at times hundreds of biological targets shown experimentally. A search combining the term “biological activity” and the name of the polyphenol, for example, returns for resveratrol 3500 results, chlorogenic acid 2000 results, and epicatechin 1500 results (Scopus searched first December 2023). I would like to call this feature of polyphenols biological promiscuity. It is a general feature of polyphenols, deeply rooted in its basic chemical properties. Polyphenols due to their multiple OH functionality and electron rich aromatic rings form multiple hydrogen bonds to protein targets accompanied by π–π interactions and π–cation interactions.^54^ We like to refer to them as protein-glue.^55,56^ When considering protein precipitation capacity as a measure for their ability to interact with proteins, excellent work by the Salminen group has elaborated two important structural features for these interactions: the number of hydrogen bond donors and the conformational flexibility of both the polyphenol and the protein target.^57^

**Figure 7:**
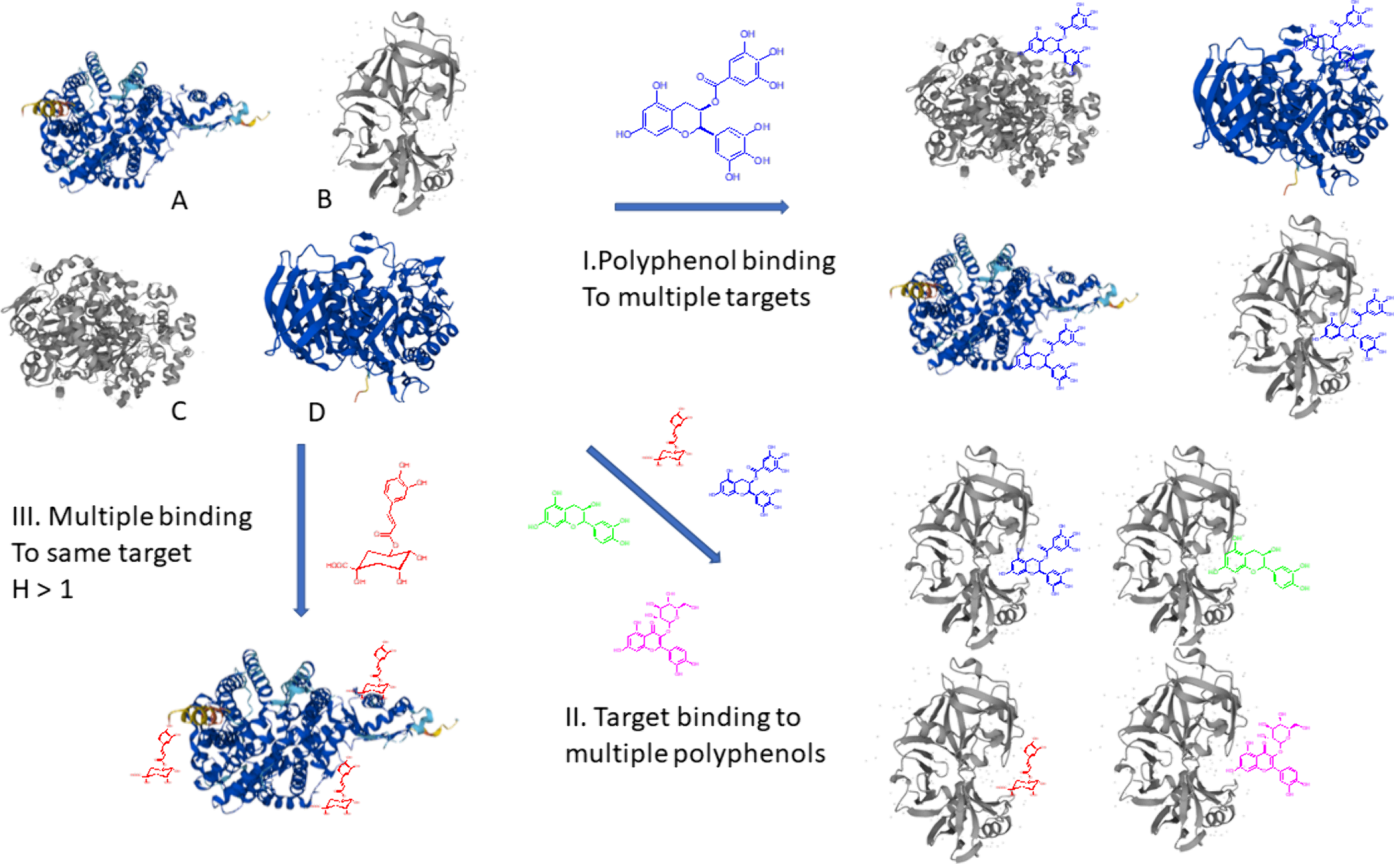
Schematic representation of biological promiscuity of polyphenols binding to protein targets (binding sites are random). Proteins structures shown taken from Uniprot (www.uniprot.org): (A) human ACE-2, (B) human lipase, (C) human α-amylase, (D) human pepsin. Polyphenols shown are 5-CQA (red), EGCG (blue), quercetin-3-glucoside (magenta), and epicatechin (green).

This biological promiscuity selectivity deficiency at multiple stages occurs at many levels illustrated schematically in Figure 7. First, a single selected polyphenol binds to multiple protein targets, second multiple polyphenols bind to the same biological target, and finally multiple polyphenols bind to a single protein target. If looking at selected prominent polyphenols, whole review articles summarize their ability to bind to multiple biological targets, however, rarely is the underlying lack of selectivity highlighted. Recent examples from the literature include resveratrol,^58^ EGCG,^59^ or chlorogenic acids.^60^

For the latter phenomenon, we observed in most polyphenol enzyme assays that the experimental Hill coefficient was larger than one. Only in rare examples does the Hill coefficient equal one. The Hill coefficient H describes the stoichiometry of the binding process, with H = 1, a single molecule mostly binding to the active site of the target and with H > 1 multiple molecules binding to the target. Such multiple allosteric binding might lead to a conformational change of the protein, often accompanied by a loss of biological function or catalytic activity of an enzyme. A subsequent literature search revealed that the large majority of Hill coefficients in the NIH high throughput screening, a database for polyphenol enzyme inhibitions showed Hill coefficient between three and five. Hence, we suggested that nonselective binding accompanied by protein denaturation and loss of function appears to be the most common mode of action for polyphenols.^61^ Similar findings were reported by the groups of Quideau^62^ and Cheynier^63^ when studying polyphenol salivary protein interactions or by ultrafiltration assays by the group of Guo identifying compounds binding to a given protein target from a multicomponent natural extract.^64,65^

In a second project, we attempted to study promiscuity by looking at general polyphenol protein affinities. Using nanodifferential fluorimetry as an experimental tool, measuring environment depending on changes of tryptophan fluorescence, avoiding protein precipitation at low concentration with fluorescence change as an indicator for binding inducing conformational change of a given protein.^66^ We screened a matrix of 12 common dietary polyphenols against 12 important protein biological targets. As a result, most polyphenols bind to 6–10 of the selected proteins. Each protein binds to 4–6 of the screened polyphenols. So again, there is promiscuity, a lack of selectivity embedded in the general biochemical properties of polyphenols. The most relevant finding here constitutes the affinity of 5-CQA to both the human ACE-2 receptor and the Coronavirus Spike protein, suggesting a protective effect against SARS Cov-2 infections of the coffee beverage.^67^ Binding affinities are always relatively high, typically between 100 μM and 5 mM, three to six orders of magnitude away from regulatory expectation for a binding constant of a to-be-approved drug molecule. However, it is worth noting that any given polyphenol never binds to all proteins or a given protein never binds to all polyphenols. There is a degree of selectivity, probably associated with polyphenol binding motifs. Selectivity occurs at a multiple target level, that is, selectivity for a whole group of proteins.

## Consequences of Findings in the Light of Screening Hypothesis and Assembly Theory

Up to this point, we have argued that polyphenol chemistry is defined by two features: reactivity leading to chemical diversity and biological promiscuity. These two features render polyphenols unsuitable for the pharmaceutical drug discovery process, drug development, and drug optimization because all of these steps command by regulatory requirements chemical and metabolic stability along with selectivity toward a chosen biological target. Nevertheless, polyphenols and plant material rich in polyphenols have undoubtedly numerous beneficial health effects. Hence the question arises: Why do reactive, unstable, and nonselective compounds benefit human health and benefit a plant in the course of evolution? We will mainly follow arguments along the line of Firn and Jones “screening hypothesis”,^68^ and the “assembly theory” proposed by Cronin,^69^ to give an answer to these questions.

According to the screening hypothesis,^70^ evolution favors organisms that could generate and retain chemical diversity at low cost. Organisms that make and “screen” a large number of chemicals will have an increased likelihood of enhanced fitness simply because the greater the chemical diversity, the greater the chances of producing the rare chemical with a useful, potent biological activity allowing survival in their ecological niche. Polyphenols fit perfectly into these requirements. By using simple and reactive building blocks branching out to more complex structures by a few both chemical and enzymatic reactions, chemical diversity is obtained at low cost. In CGA chemistry using the quinic acid as a template, diverse libraries are created by simple esterification reactions and even following biosynthesis of a given CGA, further acyl migration and epimerization allows expansion of the chemical diversity space. CGAs with their aromatic moiety and multiple H-bond donors and acceptors in different stereochemical arrangements and distances from the quinic acid core have the potential to be adapted to many possible biological targets, representing multiple pharmacophores. Examples from other plants show that additional building blocks such as hydroxy-acids or carbohydrates are used by nature to decorate and further functionalize the CGA core.^71^

Black tea chemistry is even more extreme, starting with six catechin building blocks, a library of tens of thousands of products can be obtained, for the organism some of them should turn out to be useful in obtaining a phenotype able to increase its fitness by deterring herbivores or inhibiting microbial pest organisms. It should be noted that *Camilla sinensis* is not the only plant using this strategy of producing large chemical libraries by oxidative fermentation; a similar phenomenon is observed in most cases of vegetable and fruit “browning” and found in selected medicinal plants such as *Crategus*, Umckaloabo (*Pelargonium Sidoides*), or *Cistus Incanus*.^72^

The property of biochemical promiscuity again increases the chance of finding the elusive bioactive compound. Adding a functionality with a built-in affinity for protein targets increases the chance of discovery of a useful biological functionality. Somehow, polyphenol chemistry has anticipated some major trends in medicinal chemistry, mainly combinatorial chemistry, an attempt to screen as many compounds as possible to increase the likelihood of a discovery of biological activity and fragment-based screening linking fragments with weak affinities to produce a compound with higher affinities.

Recently Lee Cronin introduced assembly theory^69^ as an additional concept attempting to quantify selection and evolution. In this theory, simple building blocks assemble to form more complex structures, with each structure being defined by its history of formation. Because the connection of building blocks, even with a limited number of possible connectivities, generates an unsustainable expansion in the number of products and possible configurations in chemical space, requiring constraints in the system for achieving evolutionary selection. Hence, assembly turns into a function of number of copies of the observed molecule and the objects assembly index. In both CGA and black tea chemistry, it becomes obvious that chemical diversity is created, but at the same time compounds with high numbers of copies are produced (sufficiently high to allow analytical detection by MS and tentative structure elucidation). We believe that this situation corresponds perfectly to Cronin’s requirement for selection, which occurs in a “transition regime” in time scales between object discovery and reproduction of the object. Accordingly, polyphenol reactivity cascades might serve as a useful model to study basic chemical evolution and selection.

## Conclusion

In conclusion, we highlighted two defining features of polyphenol chemistry. Polyphenols are highly reactive under food processing and human metabolic conditions, furnishing a myriad of novel structures and creating chemical diversity. We have illustrated these observations by presenting results from coffee chlorogenic acid and black tea chemistry. This ability to create libraries of diverse compounds in high copy numbers increases the likelihood of discovery of rare biological activity and consequently the fitness of survival of the organism according to the screening hypothesis and assembly theory. This search for biologically active compounds is accompanied by a compromise of biological promiscuity selecting and assembling selected reactive building blocks with built-in protein affinity, resulting in multiple protein binding and absence of selectivity. I hope that the findings and approaches in this assay will give thought and inspire a new generation of polyphenol scientists to solve the multitude of challenges still unresolved.
